# Transcriptome map of mouse isochores

**DOI:** 10.1186/1471-2164-12-511

**Published:** 2011-10-17

**Authors:** Stilianos Arhondakis, Kimon Frousios, Costas S Iliopoulos, Solon P Pissis, German Tischler, Sophia Kossida

**Affiliations:** 1 Bioinformatics and Medical Informatics Team, Biomedical Research Foundation of the Academy of Athens, 4 Soranou Ephessiou, 115 27, Athens, Greece; 2 Department of Informatics, King's College London, Strand, WC2R 2LS, London, UK; 3 Digital Ecosystems & Business Intelligence Institute, Centre for Stringology & Applications, Curtin University, GPO Box U1987 Perth WA 6845, Australia; 4 Department of Informatics, University of Würzburg, 97074 Würzburg, Germany

## Abstract

**Background:**

The availability of fully sequenced genomes and the implementation of transcriptome technologies have increased the studies investigating the expression profiles for a variety of tissues, conditions, and species. In this study, using RNA-seq data for three distinct tissues (brain, liver, and muscle), we investigate how base composition affects mammalian gene expression, an issue of prime practical and evolutionary interest.

**Results:**

We present the transcriptome map of the mouse isochores (DNA segments with a fairly homogeneous base composition) for the three different tissues and the effects of isochores' base composition on their expression activity. Our analyses also cover the relations between the genes' expression activity and their localization in the isochore families.

**Conclusions:**

This study is the first where next-generation sequencing data are used to associate the effects of both genomic and genic compositional properties to their corresponding expression activity. Our findings confirm previous results, and further support the existence of a relationship between isochores and gene expression. This relationship corroborates that isochores are primarily a product of evolutionary adaptation rather than a simple by-product of neutral evolutionary processes.

## Background

The genomes of vertebrates are mosaics of isochores, long regions (from 0.2Mb up to several Mb) that are fairly homogeneous in base composition. The isochores belong to a small group of families characterized by different GC levels (molar ratio of guanine and cytosine over the total number of bases of the area) [1-4]. In the human genome, a typical mammalian genome, five isochore families can be found (L1, L2, H1, H2, and H3 -- in order of increasing GC level) that cover a wide GC range (30-60%) [2-4]. The GC-richest families, H2 and H3, represent approximately 15% of the genome, and contain about 50% of the protein-coding genes. This high gene density is accompanied by other striking properties, such as open chromatin structure, localization at the center of the nucleus, high density of short interspersed elements (SINES), low density of long interspersed elements (LINES), early replication, high level of recombination, high mutation rate, and higher expression level, while GC-poorer families have the opposite properties [2]. In the mouse genome, which is of interest in this study, the L1 isochore family is under-represented, compared to other vertebrates, and the H3 family is almost absent [5]. This narrow isochore distribution in the mouse genome has been interpreted as the result of a higher substitution rate [6,7] and weak repair mechanism [8], both phenomena reducing compositional heterogeneity (see also [5]). Despite these differences, the distribution of genes is similar to that of the other vertebrates (gene density increases as GC level increases), and the average GC levels of the different families are remarkably conserved across species, reflecting a functional relation to the chromatin structure [5].

The emergence of the isochores is an open debate of relevant evolutionary importance, where in addition to the selectionist model (functional advantage [4]), other models attempt to explain the evolution of the isochores: the mutational bias [9], the GC-biased gene conversion [10,11], as also a unifying one [12]. Despite the importance of this debate, our study is focused on investigating how base composition affects mammalian gene expression. Such a relationship would provide additional evidence on a functional implication of the isochores, supporting that they are mainly a product of evolutionary adaptation [2,4], rather than a simple by-product of neutral evolutionary processes [9-11].

Previous studies have investigated the effects of base composition on gene expression, both in human and mouse tissues, through an exhaustive use of expression data from techniques based on sequencing (ESTs, SAGE, MPSS) and/or hybridization (microarrays, single-arrays, cDNA arrays) [13-21], and despite some quantitative differences, agree that the expression levels of genes are positively correlated with the GC level. Two recent studies [22,23], through *in silico *compositional analysis of expression vectors and DNA carriers, showed that aside from the GC3 level (GC level in the third codon position) of the coding sequences, the genomic compositional context in which a gene is embedded affects its expression. Additionally, the Human Transcriptome Map (HTM), using SAGE data, revealed domains of highly and weakly expressed genes [24], namely the "RIDGES" and "anti-RIDGES", respectively. The former were found to be located in gene-dense, high GC-rich, and SINE-rich genomic regions, while the latter were in regions with opposite properties [15,25]. The above reflect the partitioning of vertebrate genes into two types of genomic regions: the gene-rich regions ("genome core"), which correspond to the GC-rich isochores, and the gene-poor regions ("genome desert"), which correspond to the GC-poor isochores [2,3,26,27]. In addition, when a similar to the HTM transcriptome map was established for the mouse genome, the expression patterns were found to be conserved to that of the human genome [28,29]. Next-generation sequencing (NGS) techniques revolutionized transcriptome analyses and, compared to previous transcriptome technologies, appear to be characterized by several advantages, i.e. a better dynamic range (absence of background noise and signal saturation phenomena, although misaligned reads could be considered as background), better quantification of transcript levels and of their isoforms (absence of an upper limit to the quantification, detection of lowly expressed transcripts), identification of yet unknown coding and non-coding RNA species [30-32]. Moreover, NGS reduced the processing time and cost of sequencing by orders of magnitude, making it a more attractive tool in a broad range of research, for both DNA and RNA sequencing and for detection and analysis of genetic variability [33-36]. In this study, we took advantage of publicly available NGS data of three distinct mouse tissues [37] in order to investigate the expression patterns across the isochores of the mouse chromosomes and the effects of the isochores' compositional properties on their expression activity. In the second part, we investigated the relations between genes' expression levels and their localization in the five isochore families for the three transcriptomes considered (brain, liver, and muscle).

## Results

The results of aligning each tissue's reads to the reference mouse genome and to the coding sequences are shown in Table 1.

**Table 1:** Aligned Reads

Read data			
**Tissue**	**Total reads**	**Aligned reads**	**Reads aligned to coding sequences**

Brain	31,116,663	14,219,266	6,635,861

Liver	31,578,097	11,353,537	6,449,293

Muscle	31,763,031	14,447,075	7,931,718

Total number of reads in the dataset, number of successfully aligned reads per tissue, and number of reads aligned to coding sequences.

### The transcriptome map of the mouse isochores and the effects of their GC level on their expression activity

**Figure 1:**
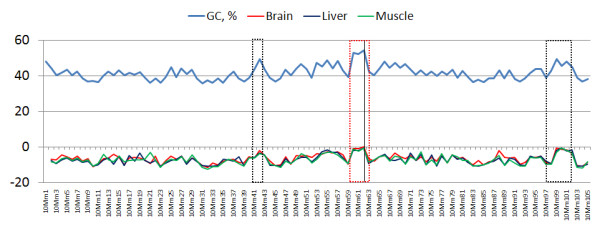
**Expression profiles of the isochore for the three tissues on chromosome 10**. The Y axis measures the isochores' GC levels (positive values -- light blue line) and their respective expression levels (*E*_*L *_-- Equation (1)) for the brain, liver, and muscle tissues (negative values -- red, dark blue, and green lines, respectively). High expression corresponds to peaks in the lines. The red and black boxes highlight areas where the high GC level is clearly accompanied by high expression. The black vertical line in the red box marks the location of the 10 Mm62 H3 isochore.

Additional file 1 shows the isochores' expression profiles for the three tissues along the whole genome, and illustrates a rough agreement of the expression levels and the GC level. One such example can be clearly seen on chromosome 10 (Figure 1). The choice of this chromosome is based on the fact that it also includes one of the very few H3 isochores of the mouse genome, the 10 Mm62 (*GC *> 53% -- marked with a vertical line in the red box in Figure 1). In the boxed areas in Figure 1, there is a clear agreement of peaks in expression and GC level, an agreement that can also be seen along most of the chromosome. To quantify this relation, we looked at the correlation between the overall expression activity of each isochore and its respective GC level, and found it to be quite strong (coefficients: *R*_*brain *_= 0.72, *R*_*liver *_= 0.62, and *R*_*muscle *_= 0.65 -- see Additional file 2).

It is well-known that in vertebrates, including the mouse, GC-richer isochores have higher gene densities compared to the GC-poorer ones (see the Background Section). This is confirmed by the positive linear correlation we found between the gene density of the isochores and their respective GC level (*R *= 0.42). Having shown the positive effect of high GC levels to the isochoric expression and between GC levels and gene density, we also looked into the direct relation between the gene density and the expression level of the individual isochores. We found a positive correlation, with similar coefficients for all tissues (coefficients: *R*_*brain *_= 0.57, *R*_*liver *_= 0.57, and *R*_*muscle *_= 0.58).

In order to isolate and investigate the effects of the GC level on the expression activity of the isochores, it was necessary to eliminate the effects of the gene density. To this end, the normalized per tissue count of reads aligned within each isochore was normalized by the respective gene density of the isochore, and the *log*_2 _values were calculated (Additional file 3). This approach limited our analysis to isochores containing at least one CDS (1, 902 isochores out of the 2, 319). As expected, we found that the percentage of isochores containing at least one CDS increased as the isochore family GC level increased (more than 60% of the L1 isochores contain no CDS against only 6% of the H2 isochores -- see Additional file 4). Notable exception to the trend is the H3 family, where an increase of isochores without any CDS is observed. However, this increasing trend in H3 isochore is due to the fact that in the mouse genome the H3 icoshores consists of just nine isochores, two of which had no CDS.

**Figure 2:**
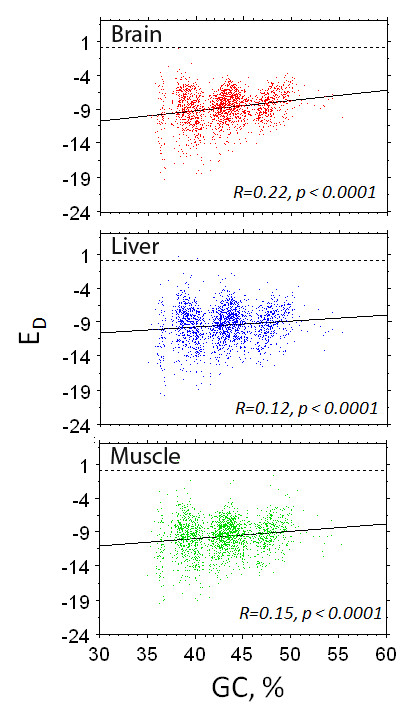
**Correlation between the solely GC effects on the expression activity of each isochore**. Correlation between the expression level (normalized by the gene density *E*_*D *_-- Equation (2)) of each isochore and the respective GC level (red plot for brain, blue plot for liver, and green plot for muscle).

We then looked at the correlation between the expression level of the isochores, normalized by the respective gene density, and their respective GC levels of the isochores, and found it to be positive for all tissues (Figure 2).

Summarizing, in this section, we initially presented the transcriptome map of the mouse isochores, and demonstrated an agreement between isochores GC level and their expression levels. Finally, after gene density effects were removed from the isochores expression levels, we found a tissue-dependent correlation between the isochores GC levels and their expression activity.

### Isochoric localization of genes and their expression activity

**Figure 3:**
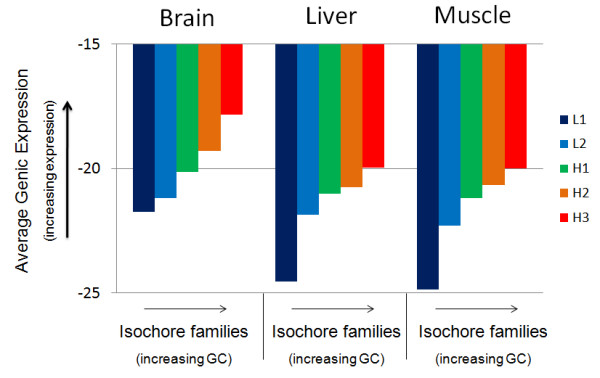
**Average genic activity within each isochore for the three tissues**. Average genic expression levels after the genes have been binned in the five isochore families. Larger negative values (tall coloured bars) indicate low expression, and small negative values (short coloured bars) indicate high expression.

In this section, we first investigated the relation between the isochoric localization of genes and their expression level. Figure 3 shows each tissue's average genic expression level per isochore family. An increase in the average genic expression can be observed as the isochore family GC level increases (statistically significant: p value < 0.001 and only 2 cases with p value < 0.01 -- Cochran test, non-parametric). The only exceptions were the differences in average genic expression between the H2 and H3 families, in the liver and muscle, and between the L1 and L2 in the brain, found to be not significant (p value > 0.05). Additionally, we found that the average genic expression of the isochore families in the brain differs significantly from that of the corresponding isochores in the muscle and liver (p value < 0.001), while between the two latter tissues significance was detected only for the L2 (p value < 0.001) and H1 families (p value < 0.005). This suggests that the expressed genes located in L1, H2, and H3 isochores in the liver and muscle appear to maintain similar expression activity.

We then looked for differences in the distribution of the expressed genes in the isochore families against that of the genes that are not expressed. As expressed, we considered genes with at least 10 aligned reads to avoid possible noise from misalignments, while as non-expressed, we considered genes without any aligned reads.

**Figure 4:**
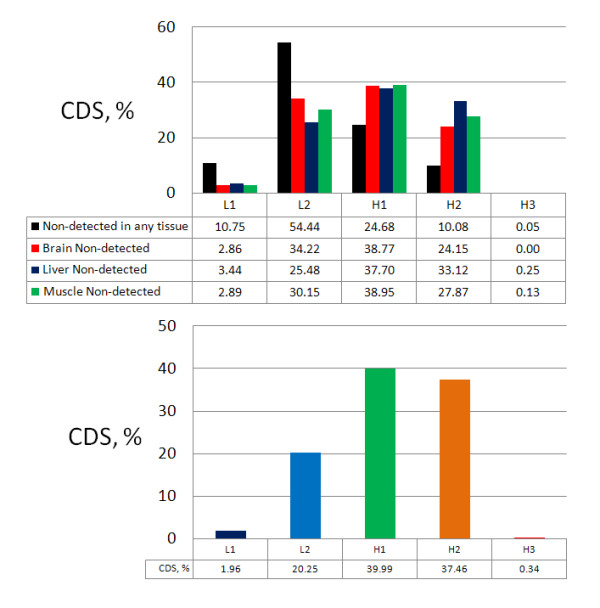
**Isochoric distributions for the non-detected genes and the total number of CDSs**. Top: Distribution (%) across the isochore families of the genes not detected to be expressed in any of the three tissues (bars in black), and of the genes not detected to be expressed in a specific tissue only (red bars for brain, blue bars for liver, and green bars for muscle). Bottom: Distribution (%) of the total number of coding sequences across the five isochore families (each coloured bar corresponds to an isochore family).

First, we identified genes that did not have detectable expression in any of the three tissues covered by the dataset (1, 925 CDSs accounting for 10.88% of the total coding sequences), and we found a very strong preference for them to be located in the L2 family (over 50% of these genes), with decreasing presence in families of subsequently higher GC (black bars in the upper panel of Figure 4). This preference for lower GC isochores is clearly different from the distribution of the total coding sequences in the isochore families (see the lower panel of Figure 4). It seems to agree with the proposition that low-GC isochores and GC-poor genes may be active during development, and are subsequently silenced in the adult stage (see the Discussion Section). For the remaining 13, 382 (15, 765 CDSs minus the 2, 383 CDSs with less 10 aligned reads), we looked into the isochoric distribution of genes that are not detected as expressed in only one of the three tissues (968 in the brain, 3, 589 in the liver, and 2, 633 in the muscle). In overall, their distribution was quite similar; centred on the H1 family, and slightly skewed towards the L1 for the brain and towards the H2 for the liver (see the upper panel of Figure 4).

Looking into the distribution of the expressed genes in the isochore families, we found no differences among the three tissues (Additional file 5). The percentage of expressed genes (12, 414 CDSs in the brain, 9, 793 in the liver, and 10, 749 in the muscle) progressively increases from low to high GC families, and peaks at the H2 family. Regarding the H3 family, the massive drop observed is related to the extreme under-representation of this family in the mouse genome. Repeating the analysis with a higher expression threshold (at least 100 reads per CDS) affects mostly the lower GC families, but overall it does not change the observed trend (data not shown). With either threshold, the distribution is different from that observed for the non-expressed genes.

In this section, we showed that genes located in GC-richer isochores have a higher expression level than genes located in GC-poor isochores. Moreover, we observed that, between liver and muscle, the genes located in L1, H2, and H3 isochores appear to maintain a similar expression activity, contrary to the expressed genes located in L2 and H1 isochores. We also presented evidence that, in three adult mouse tissues, the non-detected as expressed genes are preferably located in GC-poor isochores, while the expressed genes are preferably located in GC-rich isochores.

## Discussion

As mentioned in the Background Section, the way base composition affects mammalian gene expression is an issue of prime practical and evolutionary interest and, although it has been a matter of debate, most studies agree that there is a positive correlation. The transcriptome of the mouse isochores for the three tissues (Additional file 1, Figure 1), the positive correlation between the isochores' GC level and their respective expression activity (Figure 2), and the increase of the average expression level of genes as the GC of the isochores increases (Figure 3) support the existence of a relationship between expression level and base composition.

The herein reported correlation coefficients, between the expression activity of the isochores and their respective GC levels (Figure 2), are slightly higher to those reported in previous studies on mouse [16,19], where the genes expression was correlated with their GC3 levels. Moreover, the order in which the expression level in the three tissues is most affected by the GC level (brain > muscle > liver) agrees to those in [16]. Finally, despite the virtual absence of H3 isochores in the mouse genome and the small number of L1 isochores, our coefficients were found to be similar to those of human, the latter containing both L1 and H3 isochores [16,18-21].

In regards to the GC-poor localization of the genes that are not expressed in any of the three adult mouse tissues considered here, the notion that they may be implicated in developmental processes is supported by several studies. Indeed, two recent studies [38,39] identified, in the genome deserts of vertebrates, long-range conserved systems comprised of highly-conserved non-coding elements and their developmental regulatory gene targets. Similarly, although in a different context, it has been shown that during the development of the mouse brain, most expression changes occur in the GC-poor and LINE-rich regions [40], and that the genes expressed in the early development stages of the mouse have AT-ending codons, unlike the genes expressed in later developmental stages [41]. Genes rich in AT-ending codons are expected to be typically found in GC-poor isochore families [42].

## Conclusions

This work is the first where NGS data are used in order to establish the transcriptome map of the mouse isochores for three different tissues, and to investigate the effects of base composition on the expression activity. Our results are consistent with previous ones, and further support the idea of a functional implication of the isochores in gene expression. We conclude proposing that similar compositional approaches, using NGS data from carefully designed experiments, may shed more light into the role of the genomic (in the term of isochores) and genic compositional properties in gene expression, in the context of specific tissues or biological processes, and reveal valuable information on the implicated regulation mechanisms.

## Methods

### Data and alignment

To produce the transcriptome map of the isochores, we used publicly available RNA-seq data of three distinct mouse tissues (brain, liver, and muscle), obtained in a recent study by Mortazavi et al [37] using the standard Solexa pipeline (version 0.2.6). The initial 32-mer reads were subsequently truncated to a length of 25 base pairs. The data comes from pooled adult C57BL6 individuals. We aligned the reads against the reference mouse genome (UCSC release mm9) [43] using REad ALigner (REAL) [44,45]. REAL is based on a new, relatively simple, algorithm for the alignment of short reads onto a reference sequence. It uses two-bits-per-base encoding of the DNA alphabet for both the reference and read sequences. We used the appropriate arguments to allow up to two mismatches per read with no gaps, and to report the unique alignment with the least number of mismatches. In this case, REAL splits the reads in four fragments, and approximate string-matching implements the pigeon-hole principle [46], as a means to quickly filter out some of the alignments that have more than two mismatches. The remaining candidate alignment locations are then examined in order to eliminate the rest of them that have more than two mismatches. Unlike other current fast aligners like Bowtie [47] and SOAP2 [48], REAL is not hindered by the very short length of the reads in this dataset. This gap-less alignment method will surely miss reads that span splice sites. However, these should represent only a small fraction of the total reads. Since the study is aimed at the bigger picture, rather than the exact quantification of individual mRNAs and alternate splicing variants, the loss of sensitivity will have little impact. In any case, gapped alignment of such short single-end reads has its own perils.

### Expression level of isochores

To investigate the expression levels of the mouse isochores, the aligned reads were assigned to the isochores containing their mapped location. The locations and GC-spans of the isochores were extracted from [5]. To eliminate the effect of the different number of reads aligned from each tissue and the different length of each isochore, the aligned reads per isochore were normalized by the total count of aligned reads of the respective tissue and the length of the respective isochore. A scaling factor can be applied to lift at this stage, and then the *log*_2 _of each normalized read count was calculated as a representation of the expression level. This is represented by Equation (1), where *E*_*L *_represents the expression level normalized over the length *L *of the isochore, *R*_*i *_the read count of the isochore, *R*_*t *_the read count of the tissue, and *f *the scaling factor.

(1) $$E_{L}=\log_{2}\left(\frac{R_{i}}{R_{t}\times L}\times f\right)$$

Because the normalized counts are very small, the logarithm produces negative values, however, higher expression still corresponds to peaks. Details on the isochores' coordinates, GC levels, aligned reads, and expression levels, for each of the three tissues, can be found in Additional file 6.

As we report in the Results Section, the expression levels were also further normalized by the respective gene densities to account for the higher concentration of genes in isochores with higher GC level. If by *D *we denote the gene density of the isochore and by *E*_*D *_the isochoric expression normalized over the gene density, Equation (1) is modified as shown in Equation (2).

(2) $$E_{D}=\log_{2}\left(\frac{R_{i}}{R_{t}\times D}\times f\right)$$

### Expression level of genes

To investigate the expression at gene level, the coding sequences for the mouse were retrieved from the Consensus Coding Sequence Database (CCDS) [49]. From the 17, 704 CDSs, 14 were found to lack a starting codon, and were eliminated. The remaining 17, 690 CDSs were assigned to isochores based on the coordinates of their exons, as given in the CCDS database.

Similarly to the procedure followed for the expression levels of isochores, the expression level of a CDS (*E*_*CDS*_) was produced with Equation (3), where *R*_*CDS *_represents the count of aligned reads in the exons of each CDS, $R_{t}^{\prime }$ the total number of reads aligned to coding sequences for the tissue, and ℓ the length of the CDS.

(3) $$E_{CDS}=\log_{2}\left(\frac{R_{CDS}}{{R^{\prime }}_{t}\times \ell }\times f\right)$$

Details on the expression levels of the CDSs, for each of the three tissues, can be found in Additional file 7.

## Competing interests

The authors declare that they have no competing interests.

## Authors' contributions

SA and SK designed the study. KF, CSI, SPP, and GT processed the data, and did the computational work. SA and KF did the analysis. SA, KF, and SPP wrote the manuscript with the contribution of all authors. The final version of the manuscript is approved by all authors.

## Supplementary Material

Additional file 1 **Transcriptome profiles of the mouse isochores along the chromosomes**. The Y axis measures the isochores' GC levels (positive values -- light blue line) and their respective expression levels (*E*_*L *_-- Equation (1)) for the brain, liver, and muscle tissues (negative values -- red, dark blue, and green lines). High expression corresponds to peaks in the lines. Click here for file

Additional file 2 **Correlations between GC level and expression activity of the isochores**. The correlations between isochoric expression level (normalized over the isochoric length *E*_*L *_-- Equation (1)) and their GC. The red plot is for brain, the blue plot for liver, and the green one for muscle tissue. Click here for file

Additional file 3 **Isochoric expression levels for each tissue normalized over gene density**. This table reports the name of each isochore, the GC level (GC, %), the length (Length, Mb), the number of genes (CDS-count), the gene density (GeneDensity -- number of genes within an isochore over its length), the count of aligned reads for each tissue (Brain Count, Liver Count, and Muscle Count), the ratio between the count of aligned reads for each tissue within each isochore over the total number of reads of that tissue (#Br/TotBr, #Liv/TotLiv, and #Mus/TotMusc), and finally the isochoric expression level normalized over the gene density (LogBr(GeneDens), LogLiv(GeneDens), and LogMusc(GeneDens)). Click here for file

Additional file 4 **Distribution of the coding sequences across the five isochore families**. Within each isochore family, the % of the isochores containing at least one gene (grey bars) and of the isochores with no genes at all (light grey bars). Click here for file

Additional file 5 **Distribution of the expressed CDSs in the isochore families**. For each tissue, the % of the expressed genes (in histogram -- upper panel) within each isochore and the corresponding count (in table format -- lower panel) using as expression threshold ≥ 10 aligned reads per gene. In the histogram, the red bars indicate the genes expressed in brain, the blue bars the genes expressed in liver, and the green ones in muscle. Click here for file

Additional file 6 **Isochoric expression levels for each tissue normalized over length**. This table reports the name of each isochore, the GC level (GC, %), length (Length, Mb), the number of genes (CDS-count), the gene density (GeneDensity -- number of genes within an isochore over its length), the count of aligned reads within each isochore for each tissue (Brain Count, Liver Count, and Muscle Count), the ratio (%) between the count of aligned reads within each isochore for each tissue over the total number of reads of that tissue (#Br/TotBr, #Liv/TotLiv, and #Mus/TotMusc), and finally the global isochoric expression level normalized over the isochoric length (LogBr(Length), LogLiv(Length), and LogMusc(Length)). Click here for file

Additional file 7 **Genic expression levels for each tissue**. This table reports the isochoric localization of each coding sequence. Specifically, the first column shows the chromosome, the second indicates the isochore in which the gene is embedded, followed by its GC level and the genomic coordinates (Start (Mb) and End (Mb)). Afterwards comes the id of each coding sequence, the genomic coordinates of the coding sequence (cds_from and cds_to), the level (GC_ccds), the GC3 (GC3_ccds), the length of the coding sequence (Length_ccds), and the count of aligned reads for each tissue (brain, liver, and muscle) within each coding sequence. The three last columns report the genic expression level for each tissue (LogBr(genic), LogLiv(genic), and LogMusc(genic)). Click here for file

## References

[B1] Bernardi G Olofsson B Filipski J Zerial M Salinas J Cuny G Meunier-Rotival M Rodier F The mosaic genome of warm--blooded vertebrates Science 1985 228 953 958 10.1126/science.4001930 4001930

[B2] Bernardi G Structural and Evolutionary Genomics: Natural Selection in Genome Evolution 2005 Elsevier Science Publishers Ltd

[B3] Costantini M Clay O Auletta F Bernardi G Isochore Map of Human Chromosomes Genome Research 2006 16 536 541 10.1101/gr.4910606 16597586 PMC1457033

[B4] Bernardi G The neoselectionist Theory of Genome Evolution PNAS 2007 104 20 8385 8390 10.1073/pnas.0701652104 17494746 PMC1866311

[B5] Costantini M Cammarano R Bernardi G The evolution of isochore patterns in vertebrate genomes BMC Genomics 2008 10 146 10.1186/1471-2164-10-146 PMC2678159 19344507

[B6] Wu C Li W Evidence for higher rates of nucleotide substitution in rodents than in man PNAS 1985 82 1741 1745 10.1073/pnas.82.6.1741 3856856 PMC397348

[B7] Gu X Li W Higher rates of amino acids substitution in rodents than in human Mol Phylogenet Evol 1992 1 211 214 10.1016/1055-7903(92)90017-B 1342937

[B8] Holliday R Understanding Ageing 1995 Cambridge University Press, Cambridge, U.K

[B9] Eyre-Walker A Hurst LD The evolution of isochores Nature Reviews Genetics 2001 2 7 549 555 10.1038/35080577 11433361

[B10] Galtier N Piganeau G Mouchiroud D Duret L GC-Content Evolution in Mammalian Genomes: The Biased Gene Conversion Hypothesis Genetics 2001 159 2 907 911 11693127 10.1093/genetics/159.2.907 PMC1461818

[B11] Duret L Galtier N Biased Gene Conversion and the Evolution of Mammalian Genomic Landscapes Annual Review of Genomics and Human Genetics 2009 10 285 311 10.1146/annurev-genom-082908-150001 19630562

[B12] Chojnowski J Franklin J Katsu Y Patterns of Vertebrate Isochore Evolution Revealed by Comparison of Expressed Mammalian, Avian, and Crocodilian Genes Journal of Molecular Evolution 2007 65 3 259 266 10.1007/s00239-007-9003-2 17674077

[B13] Duret L Evolution of synonymous codon usage in metazoans Current Opinion in Genetics & Development 2002 12 6 640 649 10.1016/S0959-437X(02)00353-2 12433576

[B14] Konu O Li M Correlations between mRNA expression levels and GC contents of coding and untranslated regions of genes in rodents Journal of Molecular Evolution 2002 54 35 41 10.1007/s00239-001-0015-z 11734896

[B15] Versteeg R van Schaik B van Batenburg M The human transcriptome map reveals extremes in gene dentistry, intron length, GC content, and repeat pattern for domains of highly and weakly expressed genes Genome Research 2003 13 9 1998 2004 10.1101/gr.1649303 12915492 PMC403669

[B16] Vinogradov A Isochores and tissue specificity Nucleic Acids Research 2003 31 17 5212 5220 10.1093/nar/gkg699 12930973 PMC212799

[B17] Arhondakis S Auletta F Torelli G D'Onofrio G Base composition and expression level of human genes Gene 2004 325 165 169 14697521 10.1016/j.gene.2003.10.009

[B18] Comeron J Selective and Mutational Patterns Associated With Gene Expression in Humans: Influences on Synonymous Composition and Intron Presence Genetics 2004 167 3 1293 1304 10.1534/genetics.104.026351 15280243 PMC1470943

[B19] Semon M Mouchiroud D Duret L Relationship between gene expression and GC-content in mammals: statistical significance and biological relevance Human Molecular Genetics 2005 14 3 421 427 15590696 10.1093/hmg/ddi038

[B20] Vinogradov A Dualism of gene GC content and CpG pattern in regard to expression in the human genome: Magnitude versus breadth Trends in Genetics 2005 21 12 639 643 10.1016/j.tig.2005.09.002 16202472

[B21] Arhondakis S Clay O Bernardi G Compositional properties of human cDNA libraries: Practical implications FEBS Letters 2006 580 24 5772 5778 10.1016/j.febslet.2006.09.034 17022979

[B22] Arhondakis S Clay O Bernardi G GC level and expression of human coding sequences Biochemical and Biophysical Research Communications 2008 367 3 542 545 10.1016/j.bbrc.2007.12.155 18177737

[B23] Mahmud A Amore G Bernardi G Compositional Genome Contexts Affect Gene Expression Control in Sea Urchin Embryo PLoS ONE 2008 3 12 e4025 10.1371/journal.pone.0004025 19112499 PMC2603317

[B24] Caron H van Schaik B van der Mee M The Human Transcriptome Map: Clustering of Highly Expressed Genes in Chromosomal Domains Science 2001 291 5507 1289 1292 10.1126/science.1056794 11181992

[B25] Lercher M Urrutia A Pavlicek A Hurst L A unification of mosaic structures in the human genome Human Molecular Genetics 2003 12 19 2411 2415 10.1093/hmg/ddg251 12915446

[B26] Mouchiroud D D'Onofrio G Aissani B The distribution of genes in the human genome Gene 1991 100 181 187 2055469 10.1016/0378-1119(91)90364-h

[B27] Zoubak S Clay O Bernardi G The gene distribution of the human genome Gene 1996 174 95 102 10.1016/0378-1119(96)00393-9 8863734

[B28] Mijalski T Harder A Halder T Identification of coexpressed gene clusters in a comparative analysis PNAS 2005 102 24 8621 8626 10.1073/pnas.0407672102 15939889 PMC1143582

[B29] Singer G Lloyd A Huminiecki L Wolfe K Clusters of Co-expressed Genes in Mammalian Genomes Are Conserved by Natural Selection Molecular Biology and Evolution 2005 22 3 767 775 15574806 10.1093/molbev/msi062

[B30] Wang Z Gerstein M Snyder M RNA-seq: a revolutionary tool for transcriptomics Nature Reviews Genetics 2009 10 57 63 10.1038/nrg2484 19015660 PMC2949280

[B31] Metzker M Sequencing technologies -- the next generation Nature Reviews Genetics 2010 11 31 46 10.1038/nrg2626 19997069

[B32] Ozsolak F Milos P RNA sequencing: advances, challenges and opportunities Nature Reviews Genetics 2011 12 2 87 98 10.1038/nrg2934 21191423 PMC3031867

[B33] Dalca A Brudno M Genome variation discovery with high-throughput sequencing data Briefings in Bioinformatics 2010 11 bbp058 14 10.1093/bib/bbp058 20053733

[B34] Ng S Buckingham K Lee C Exome sequencing identifies the cause of a mendelian disorder Nature Genetics 2010 42 30 35 10.1038/ng.499 19915526 PMC2847889

[B35] Wu T Nacu S Fast and SNP--tolerant detection of complex variants and splicing in short reads Bioinformatics 2010 26 7 873 881 10.1093/bioinformatics/btq057 20147302 PMC2844994

[B36] Xiang H Zhu J Chen Q Single--base resolution methylome of the silkworm reveals a sparse epigenomic map Nature Biotechnology 2010 28 5 516 520 10.1038/nbt.1626 20436463

[B37] Mortazavi A Williams B McCue K Mapping and quantifying mammalian transcriptomes by RNA-seq Nature Methods 2008 5 7 621 628 10.1038/nmeth.1226 18516045 PMC13303166

[B38] Kikuta H Laplante M Navratilova P Genomic regulatory blocks encompass multiple neighboring genes and maintain conserved synteny in vertebrates Genome Research 2007 17 5 545 555 10.1101/gr.6086307 17387144 PMC1855176

[B39] Navratilova P Becker T Genomic regulatory blocks in vertebrates and implications in human disease Briefings in Functional Genomics & Proteomics 2009 8 4 333 342 10.1093/bfgp/elp019 19561171

[B40] Hiratani I Leskovar A Gilbert D Differentiation--induced replication-timing changes are restricted to AT--rich/long interspersed nuclear element (LINE)--rich isochores Proceedings of the National Academy of Sciences of the United States of America 2004 101 48 16861 16866 10.1073/pnas.0406687101 15557005 PMC534734

[B41] Ren L Gao G Zhao D Developmental stage related patterns of codon usage and genomic GC content: Searching for evolutionary fingerprints with models of stem cell differentiation Genome Biology 2007 8 3 10.1186/gb-2007-8-3-r35 PMC1868930 17349061

[B42] Clay O Bernardi G GC3 of Genes Can Be Used as a Proxy for Isochore Base Composition: A Reply to Elhaik et al Molecular Biology and Evolution 2011 28 21 23 10.1093/molbev/msq222 20817719

[B43] UCSC Genome Browser 2011 http://genome.ucsc.edu

[B44] Frousios K Iliopoulos CS Mouchard L REAL: an efficient REad ALigner for next generation sequencing reads Proceedings of the First ACM International Conference on Bioinformatics and Computational Biology 2010 New York, NY, USA: ACM 154 159 BCB '10,

[B45] REad ALigner (REAL) 2011 http://www.inf.kcl.ac.uk/pg/real/

[B46] Navarro G Raffinot M Flexible Pattern Matching in Strings: Practical On-Line Search Algorithms for Texts and Biological Sequences 2002 Cambridge University Press

[B47] Langmead B Trapnell C Pop M Salzberg S Ultrafast and memory--efficient alignment of short DNA sequences to the human genome Genome Biology 2009 10 3 R25+ 19261174 10.1186/gb-2009-10-3-r25 PMC2690996

[B48] Li R Li Y Kristiansen K Wang J SOAP: short oligonucleotide alignment program Bioinformatics 2008 24 5 btn025 714 10.1093/bioinformatics/btn025 18227114

[B49] National Center for Biotechnology Information (NCBI) 2011 ftp://ftp.ncbi.nlm.nih.gov

