# Supplementary figures and images for: Transcriptome map of mouse isochores
Source: BMC Genomics. 2011 Oct 17;12:511. doi: 10.1186/1471-2164-12-511 (PMC3215772; doi:10.1186/1471-2164-12-511)

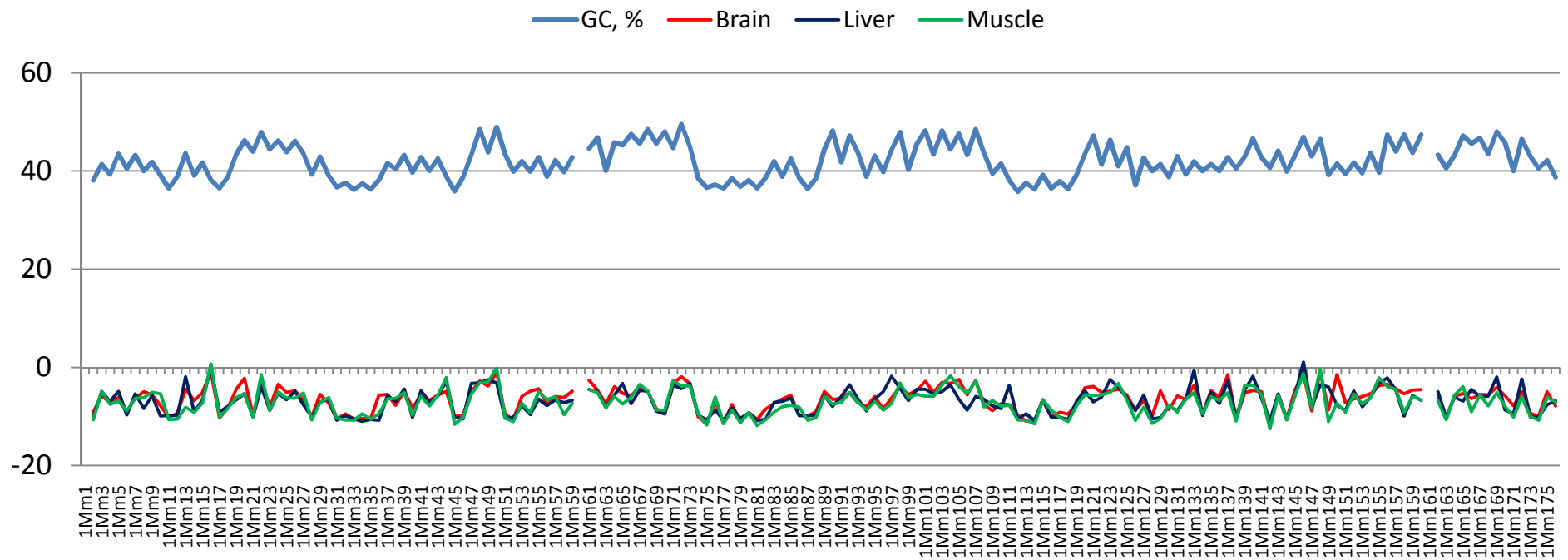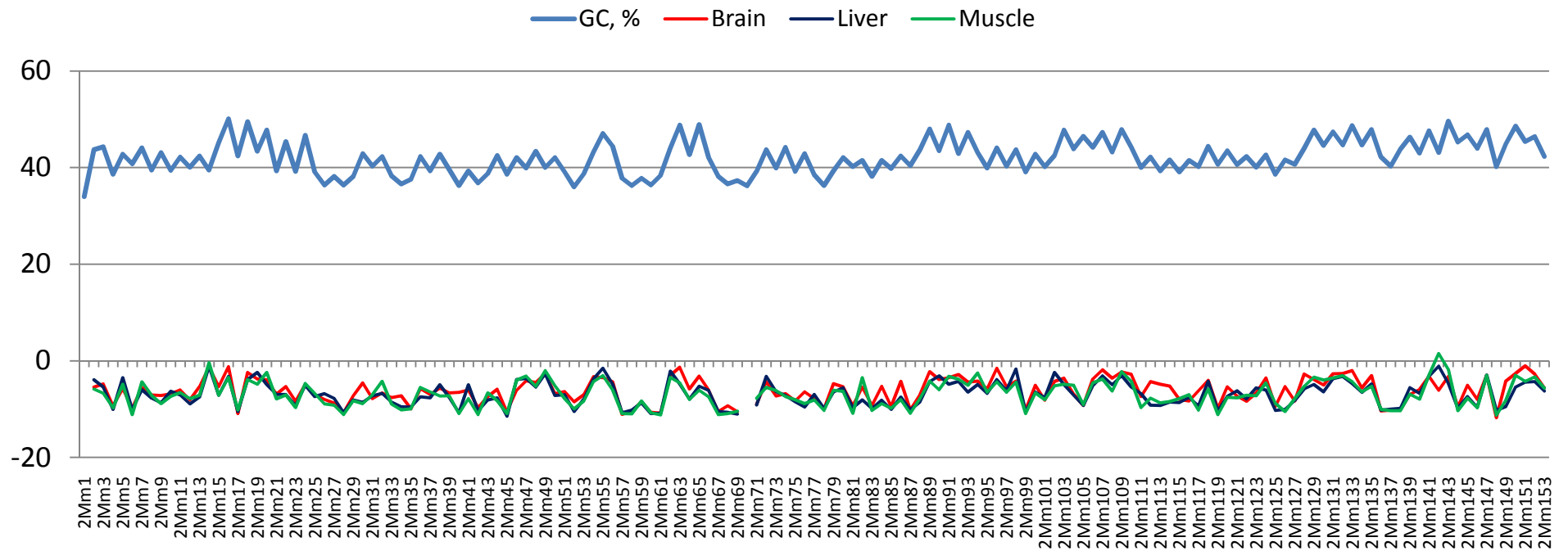

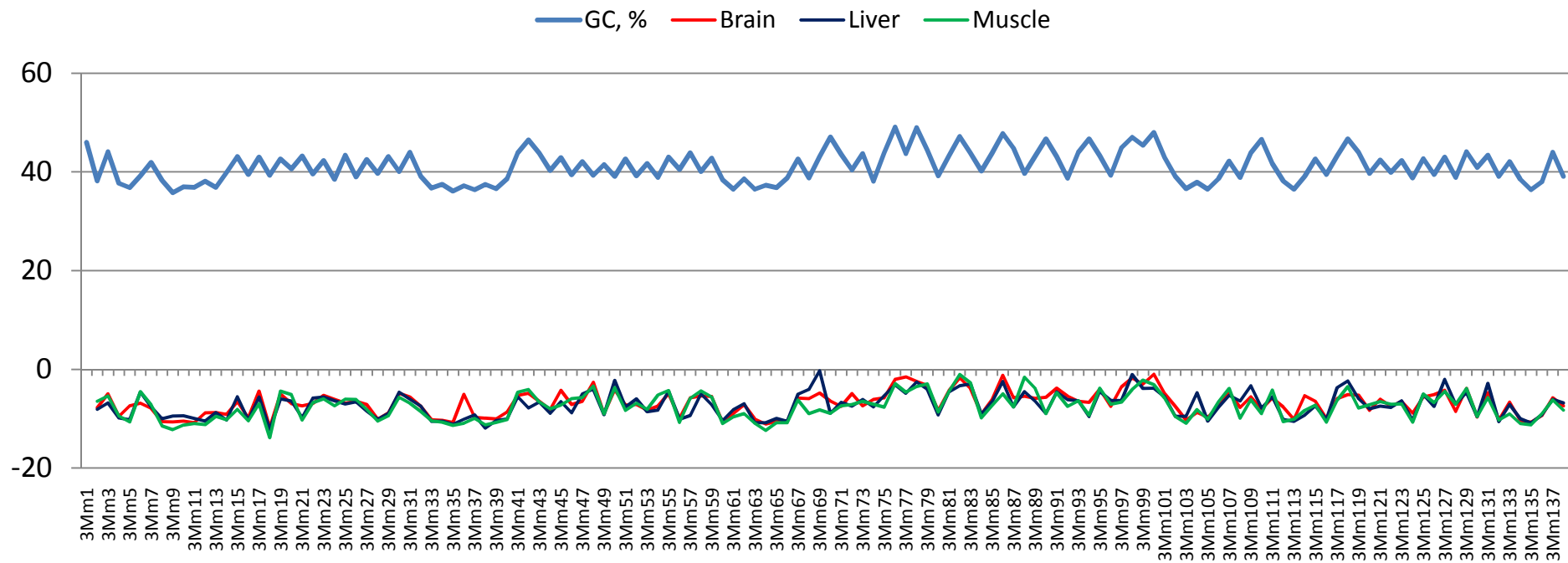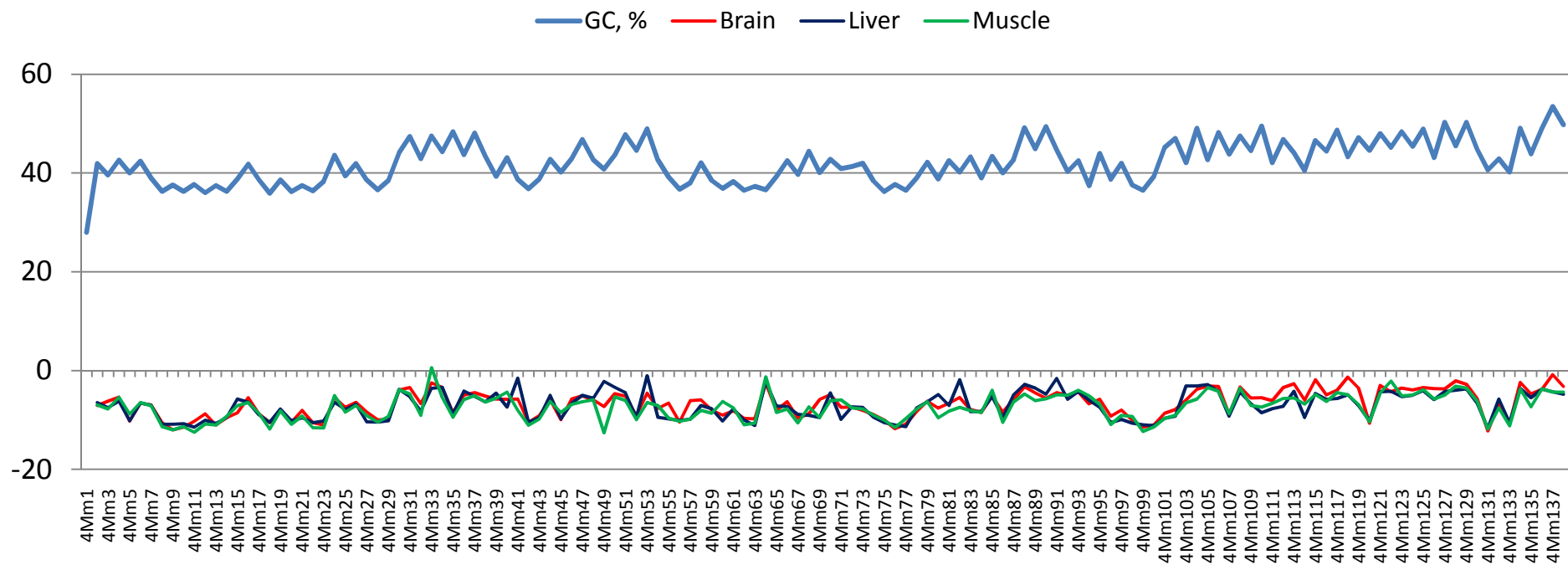

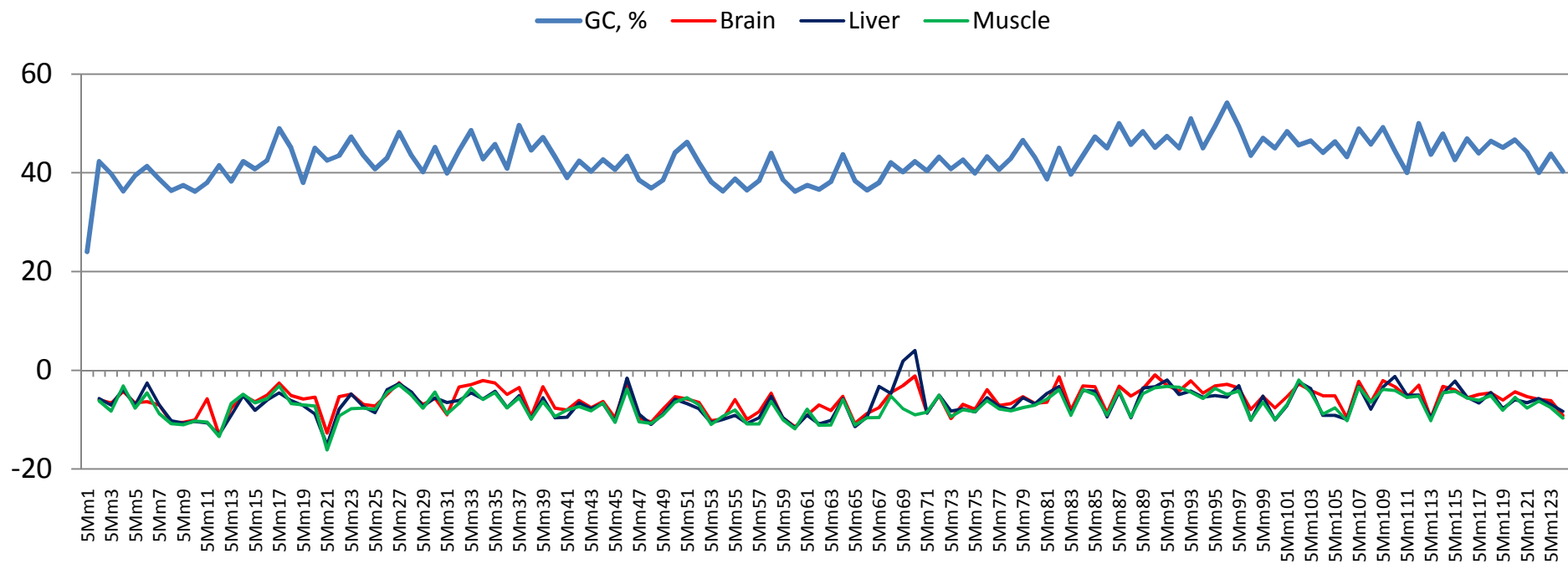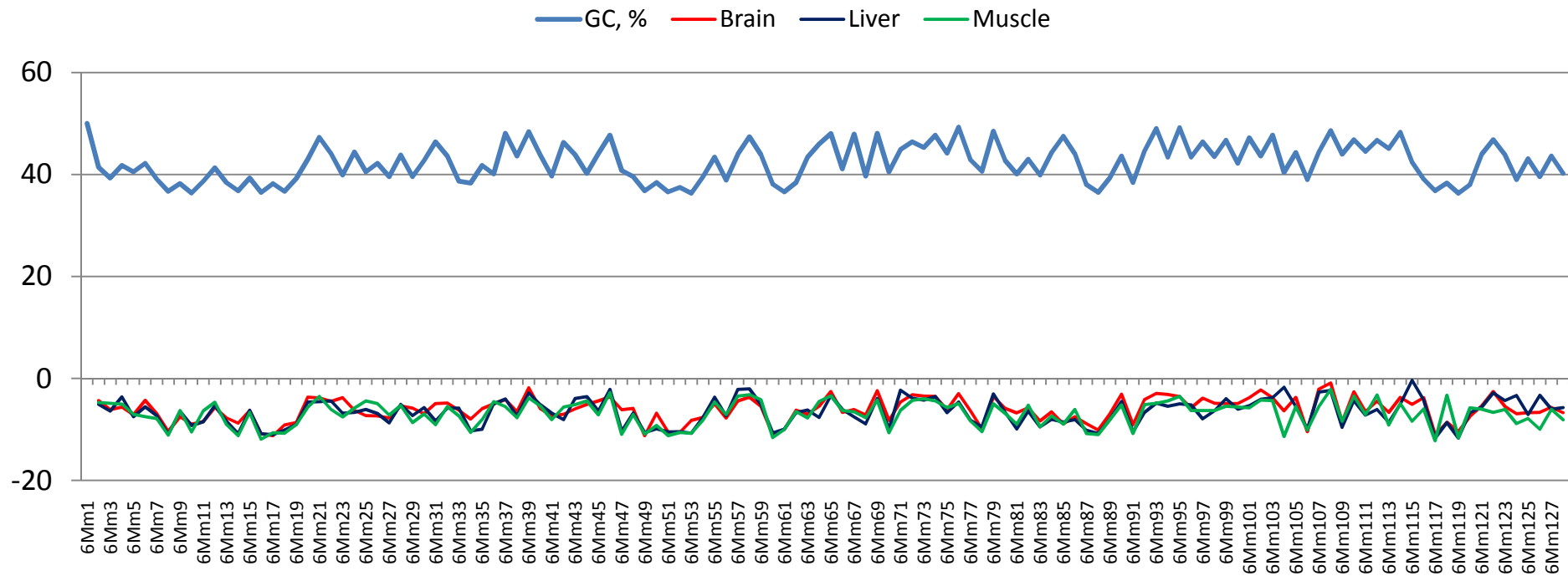

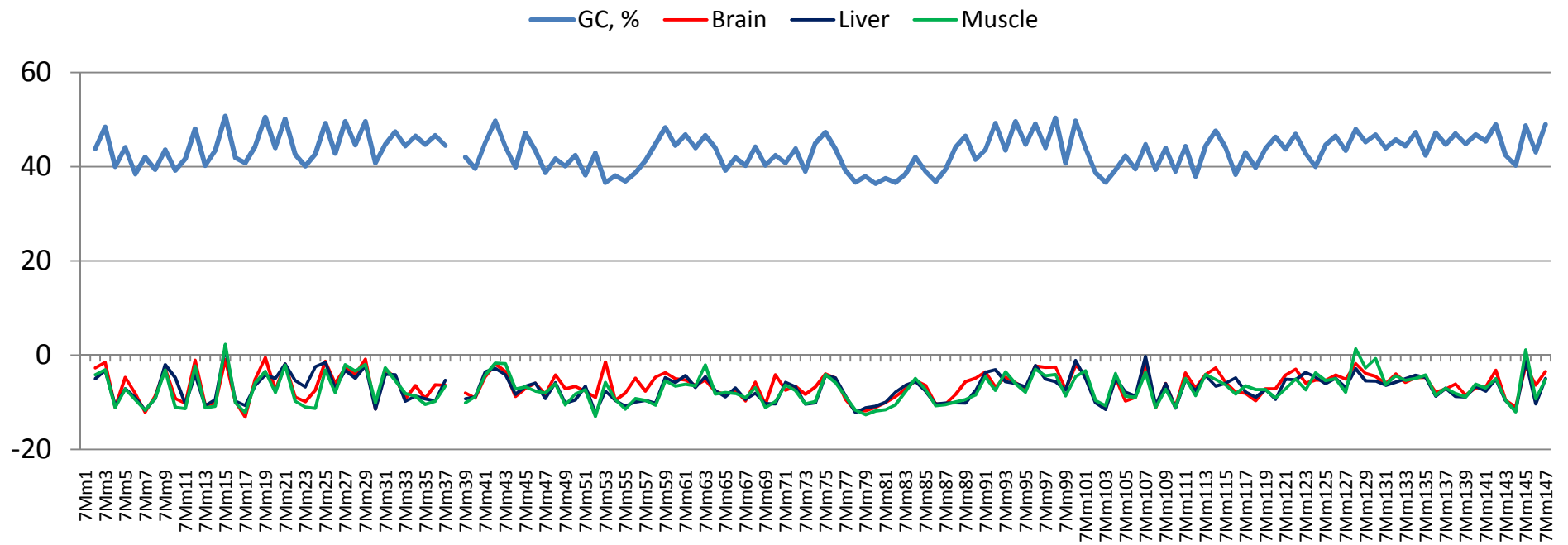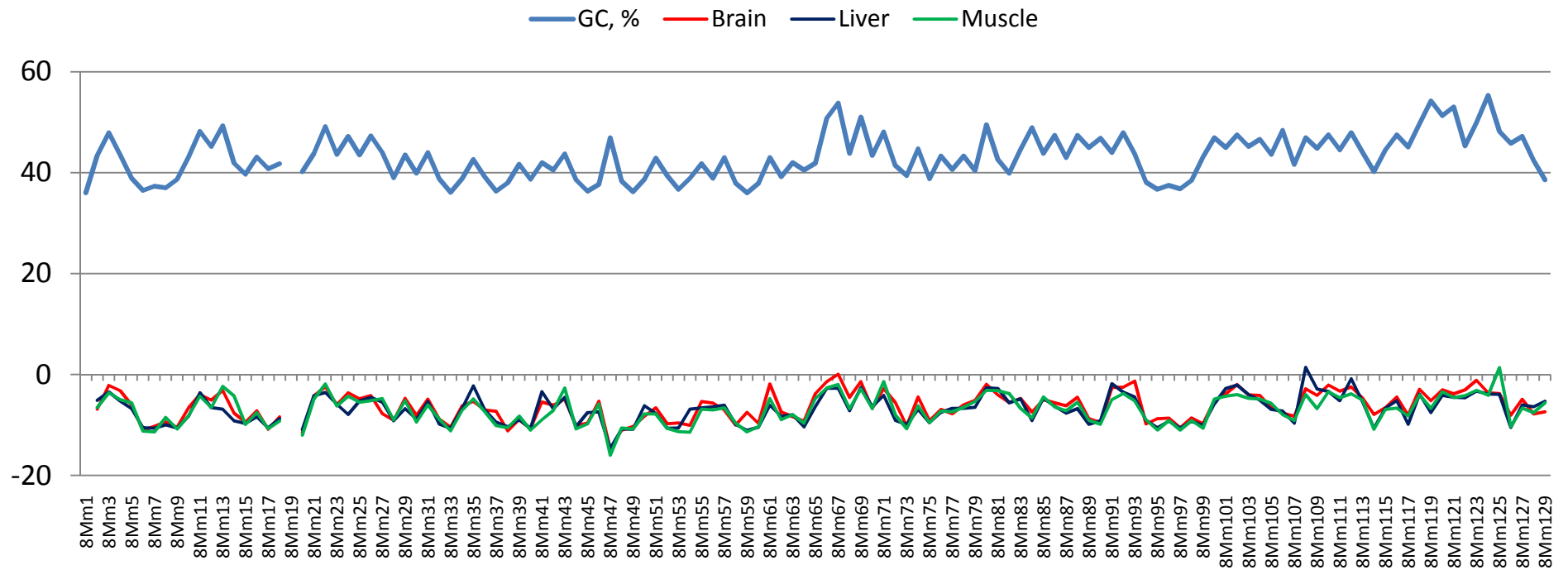

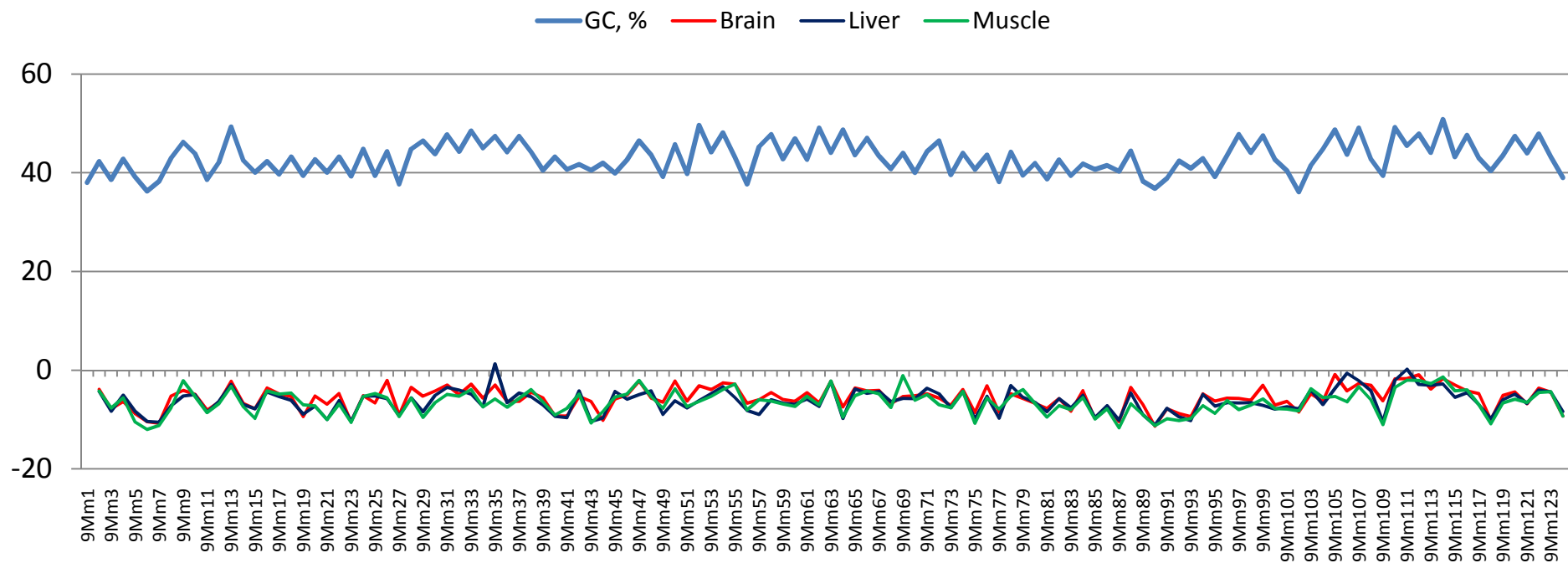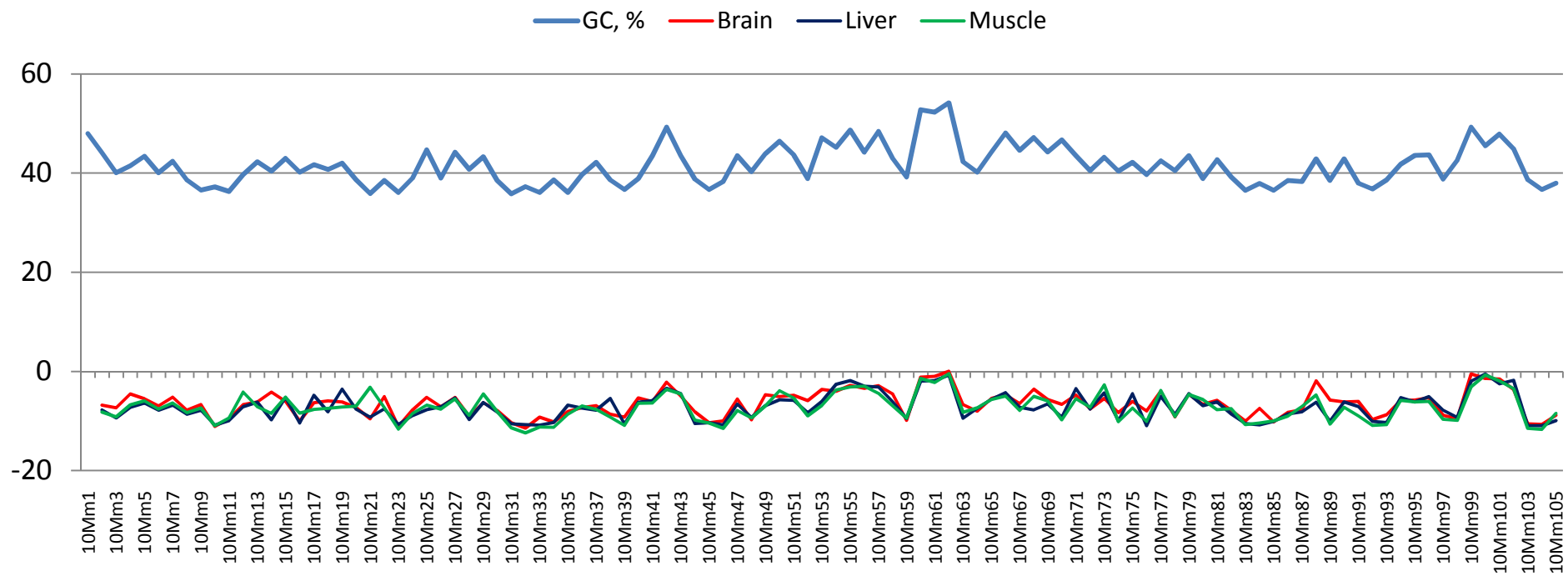

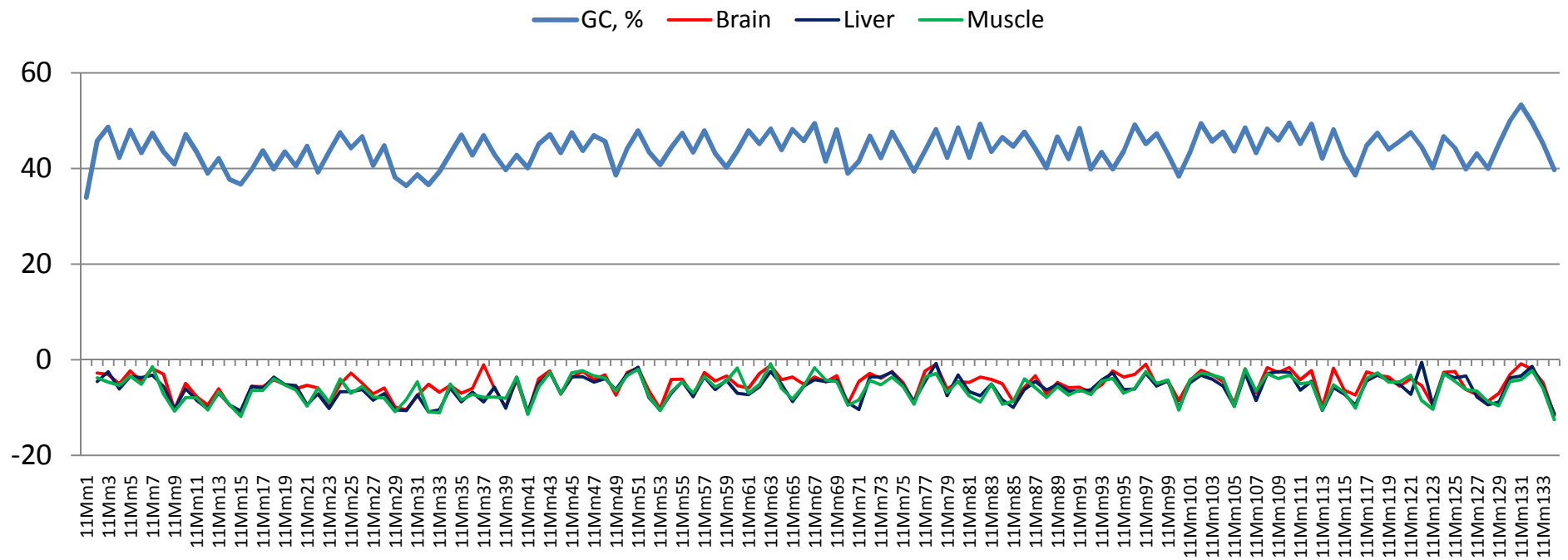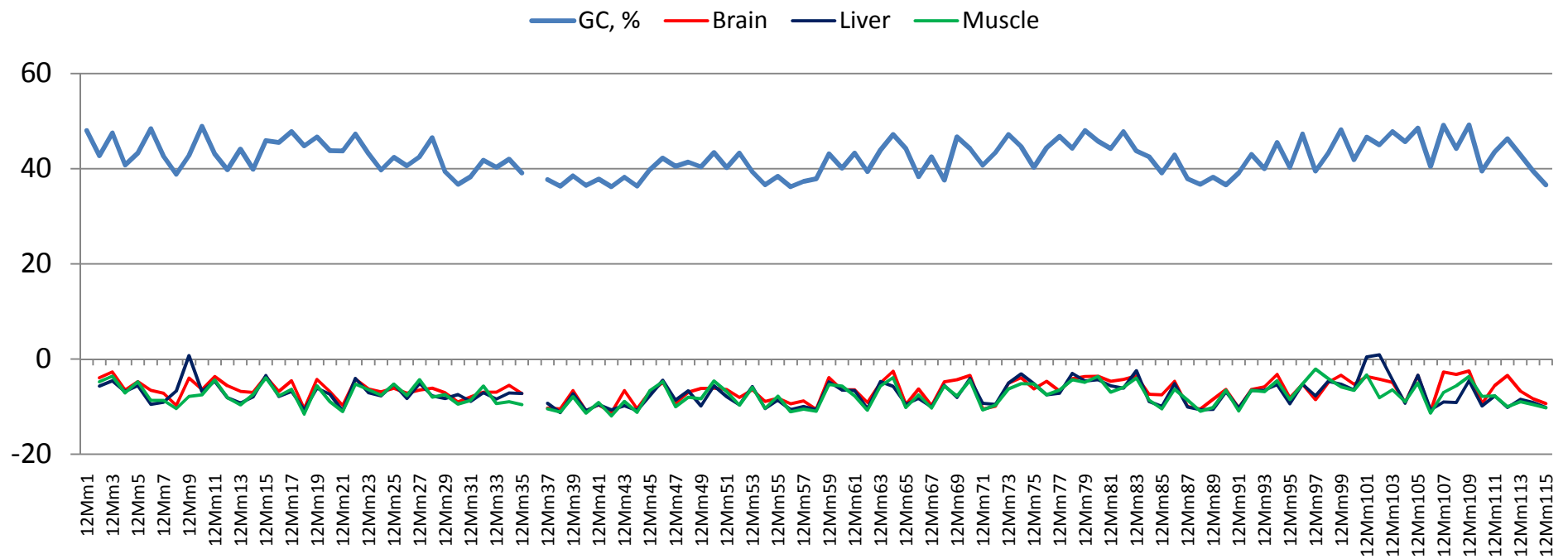

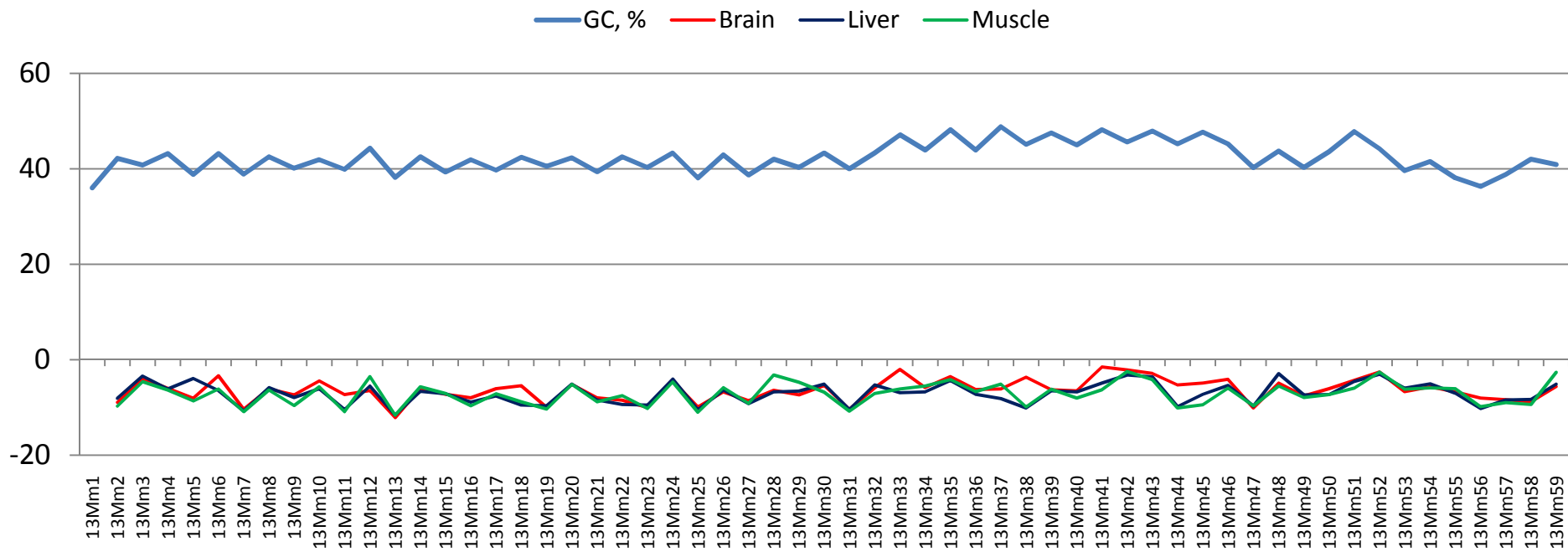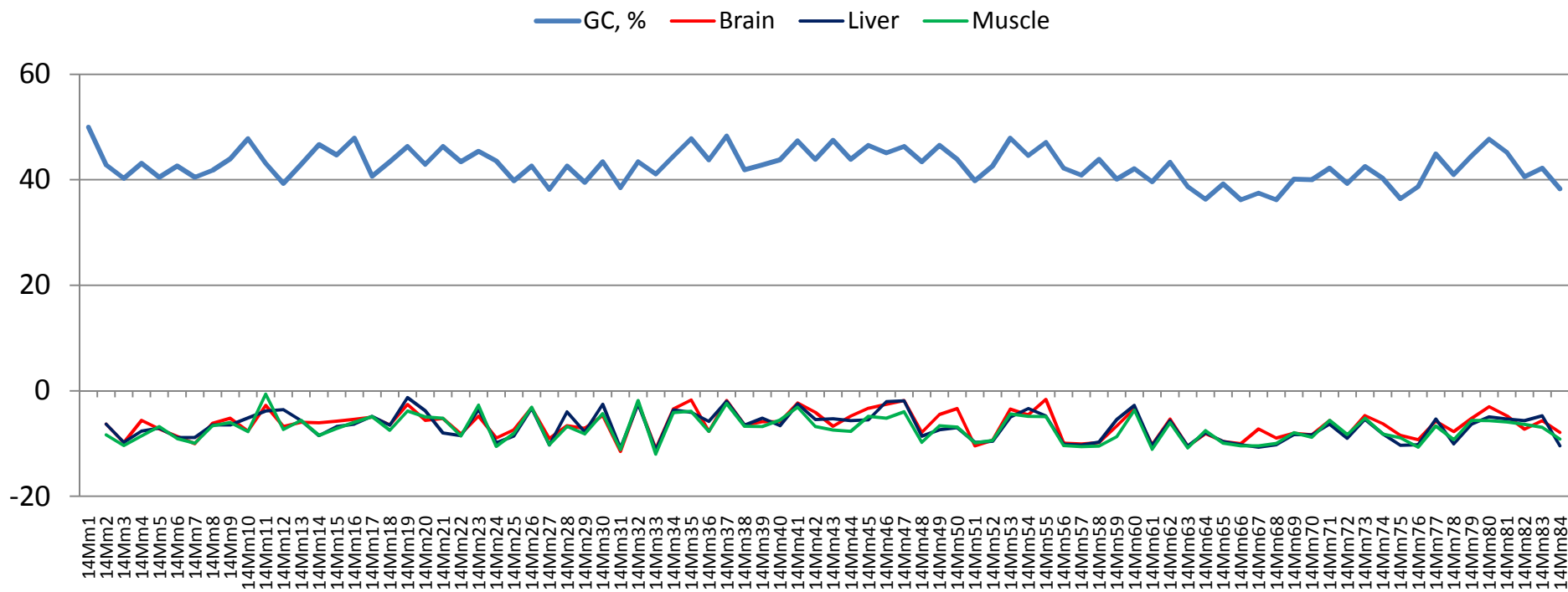

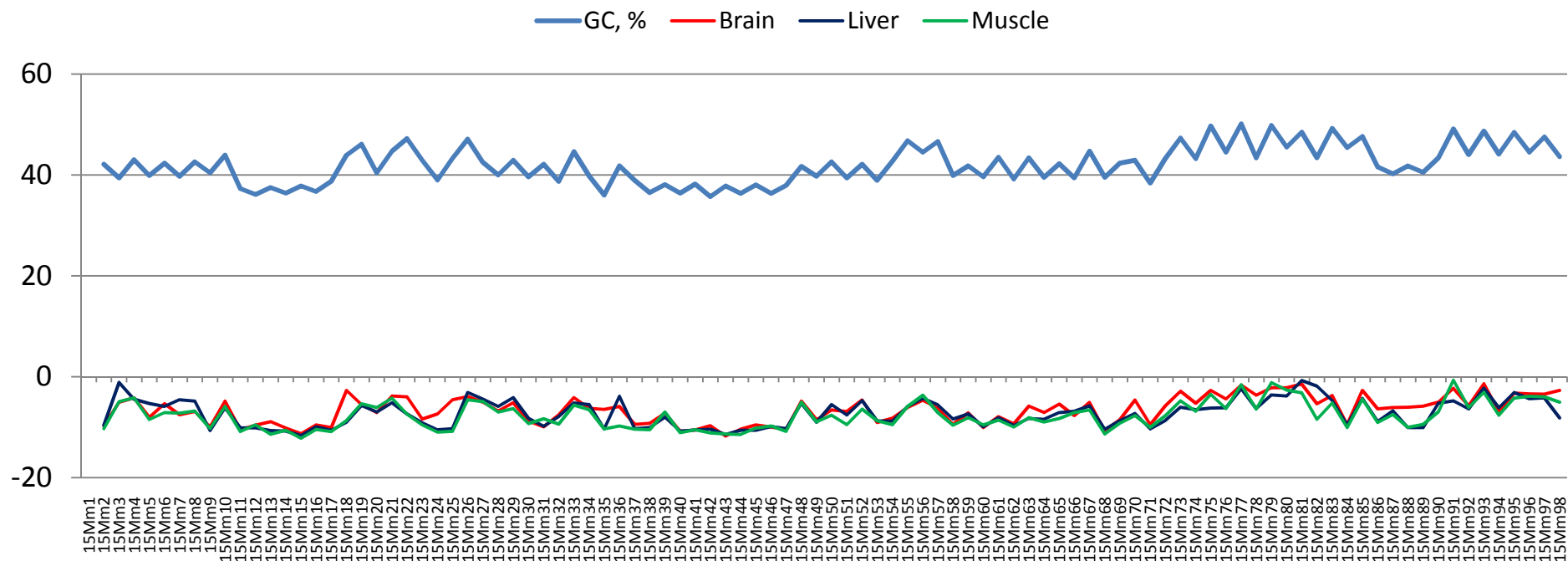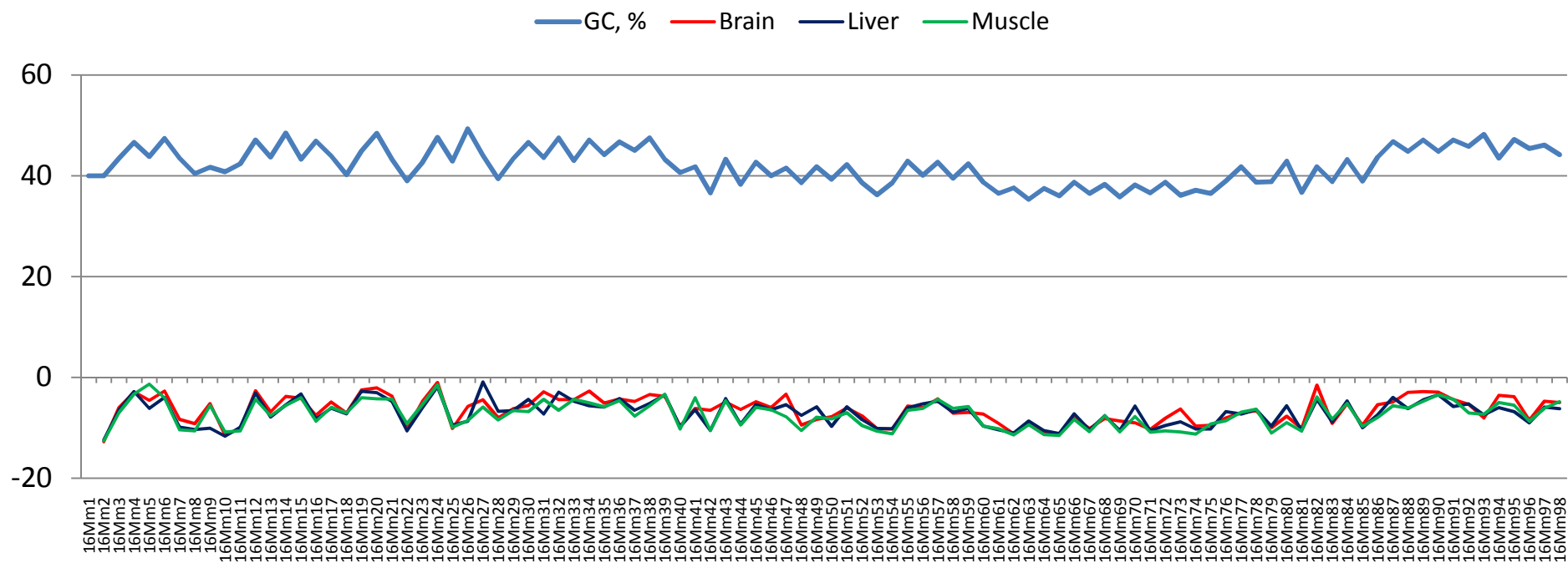

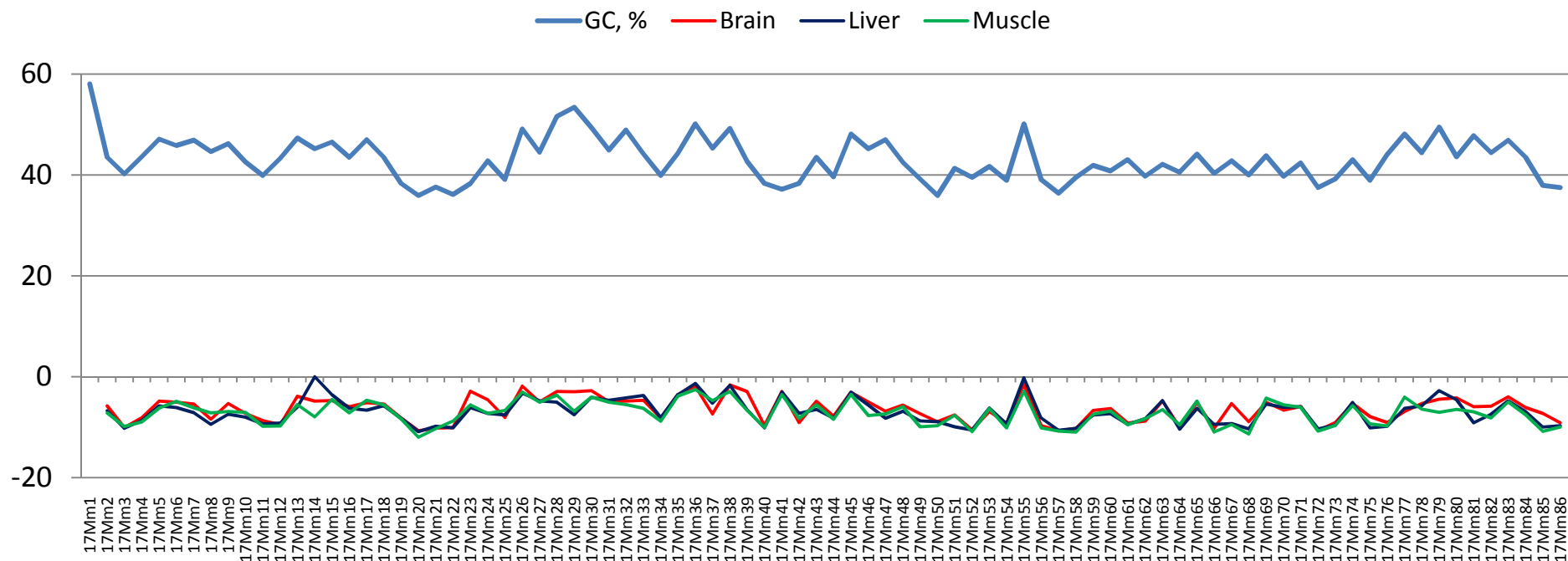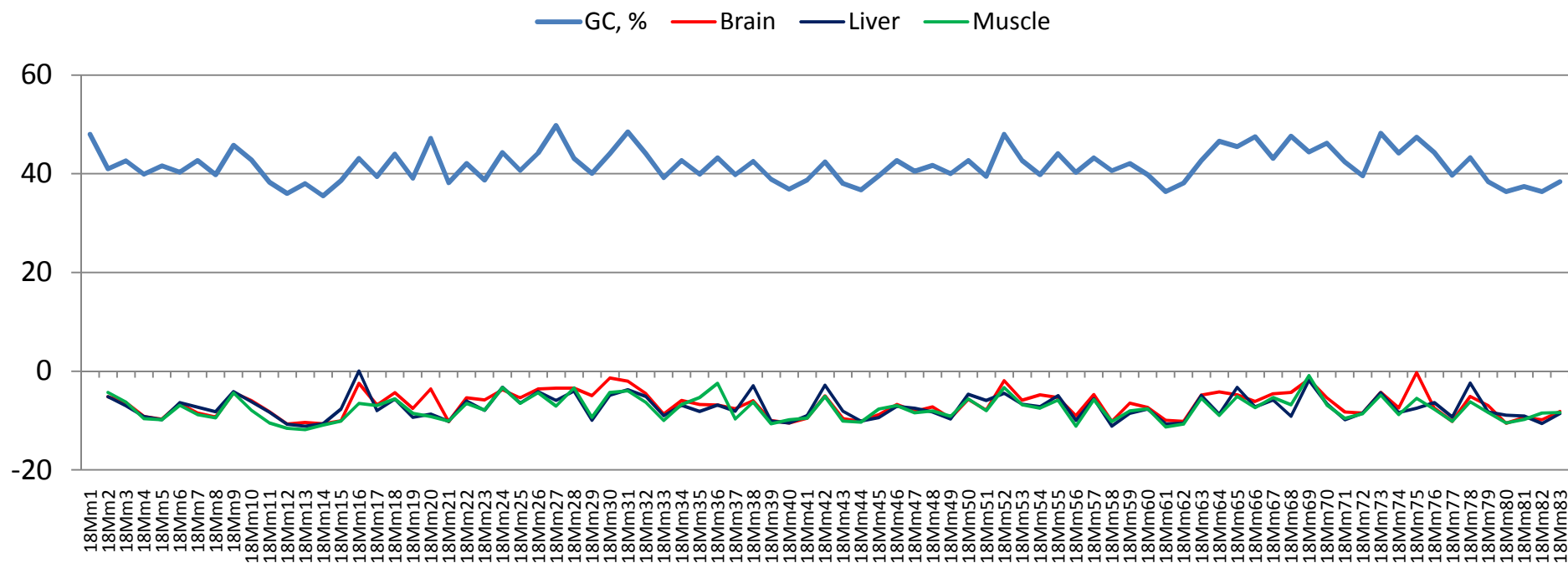

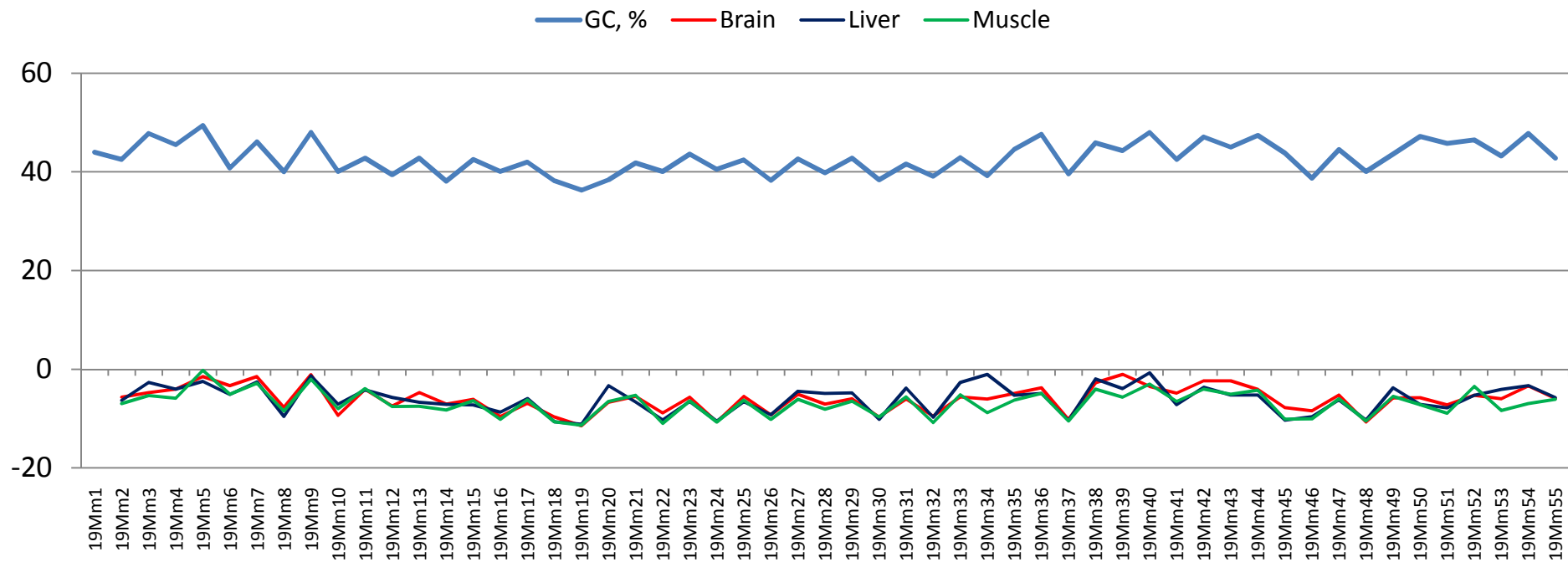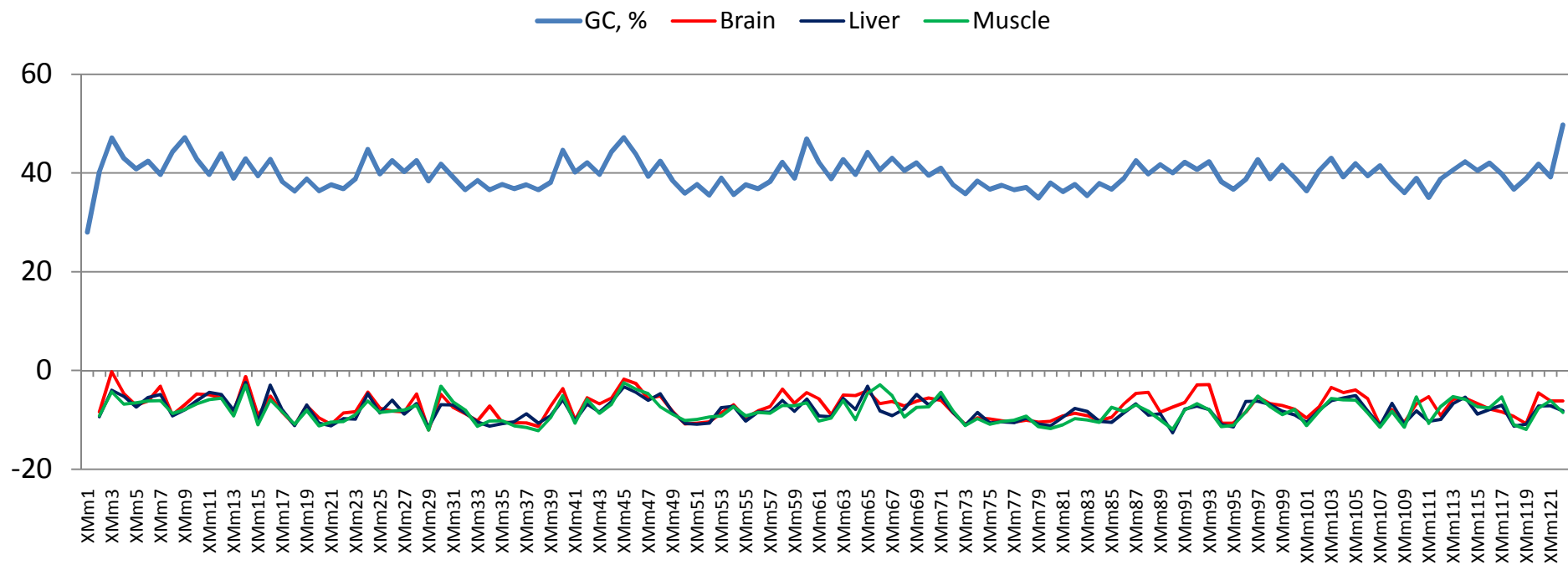

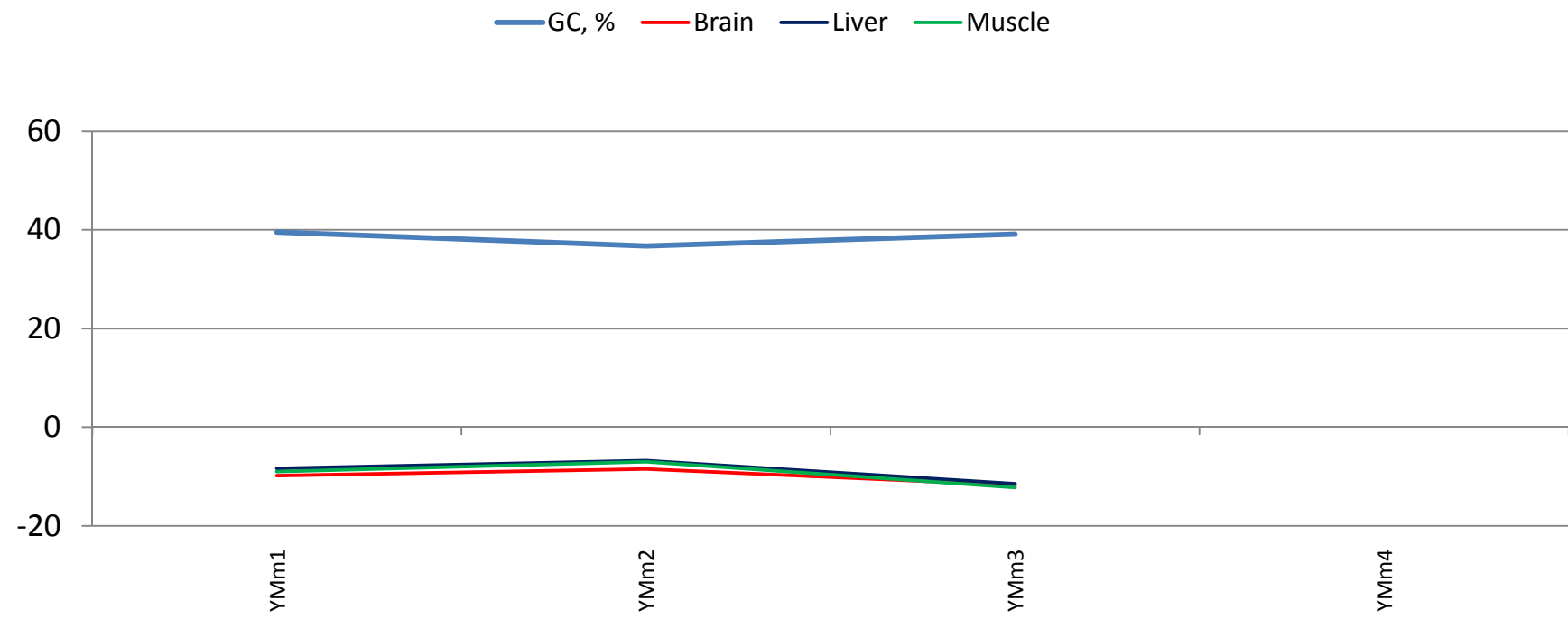

Supplement: Additional file 1 — Transcriptome profiles of the mouse isochores along the chromosomes. The Y axis measures the isochores' GC levels (positive values -- light blue line) and their respective expression levels (EL -- Equation (1)) for the brain, liver, and muscle tissues (negative values -- red, dark blue, and green lines). High expression corresponds to peaks in the lines. [file 1471-2164-12-511-S1.PDF]

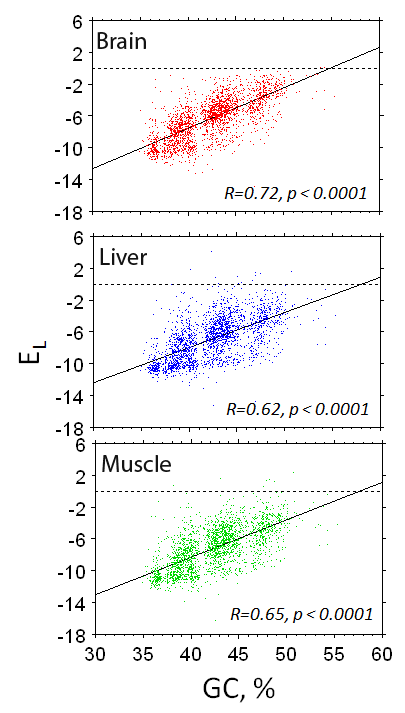

Supplement: Additional file 2 — Correlations between GC level and expression activity of the isochores. The correlations between isochoric expression level (normalized over the isochoric length EL -- Equation (1)) and their GC. The red plot is for brain, the blue plot for liver, and the green one for muscle tissue. [file 1471-2164-12-511-S2.TIFF]

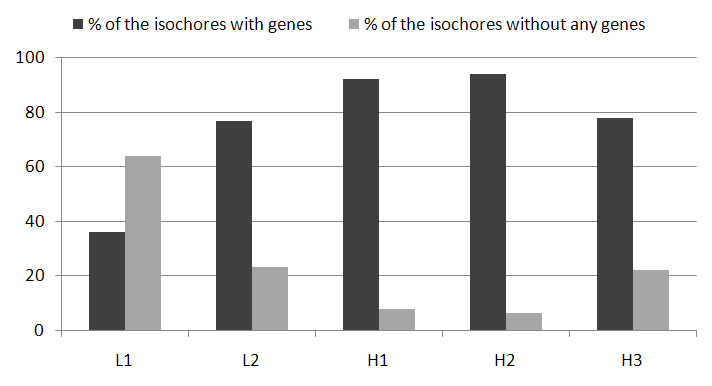

Supplement: Additional file 4 — Distribution of the coding sequences across the five isochore families. Within each isochore family, the % of the isochores containing at least one gene (grey bars) and of the isochores with no genes at all (light grey bars). [file 1471-2164-12-511-S4.TIFF]

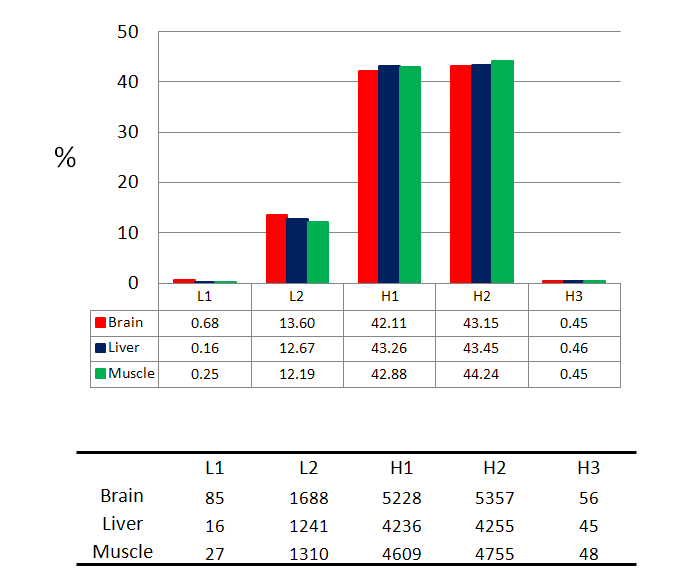

Supplement: Additional file 5 — Distribution of the expressed CDSs in the isochore families. For each tissue, the % of the expressed genes (in histogram -- upper panel) within each isochore and the corresponding count (in table format -- lower panel) using as expression threshold ≥ 10 aligned reads per gene. In the histogram, the red bars indicate the genes expressed in brain, the blue bars the genes expressed in liver, and the green ones in muscle. [file 1471-2164-12-511-S5.TIFF]
